# Marker density and statistical model designs to increase accuracy of genomic selection for wool traits in Angora rabbits

**DOI:** 10.3389/fgene.2022.968712

**Published:** 2022-09-02

**Authors:** Chao Ning, Kerui Xie, Juanjuan Huang, Yan Di, Yanyan Wang, Aiguo Yang, Jiaqing Hu, Qin Zhang, Dan Wang, Xinzhong Fan

**Affiliations:** College of Animal Science and Veterinary Medicine, Shandong Agricultural University, Tai’an, China

**Keywords:** angora rabbit, wool, genomic selection, marker density, model

## Abstract

The Angora rabbit, a well-known breed for fiber production, has been undergoing traditional breeding programs relying mainly on phenotypes. Genomic selection (GS) uses genomic information and promises to accelerate genetic gain. Practically, to implement GS in Angora rabbit breeding, it is necessary to evaluate different marker densities and GS models to develop suitable strategies for an optimized breeding pipeline. Considering a lack in microarray, low-coverage sequencing combined with genotype imputation was used to boost the number of SNPs across the rabbit genome. Here, in a population of 629 Angora rabbits, a total of 18,577,154 high-quality SNPs were imputed (imputation accuracy above 98%) based on low-coverage sequencing of 3.84X genomic coverage, and wool traits and body weight were measured at 70, 140 and 210 days of age. From the original markers, 0.5K, 1K, 3K, 5K, 10K, 50K, 100K, 500K, 1M and 2M were randomly selected and evaluated, resulting in 50K markers as the baseline for the heritability estimation and genomic prediction. Comparing to the GS performance of single-trait models, the prediction accuracy of nearly all traits could be improved by multi-trait models, which might because multiple-trait models used information from genetically correlated traits. Furthermore, we observed high significant negative correlation between the increased prediction accuracy from single-trait to multiple-trait models and estimated heritability. The results indicated that low-heritability traits could borrow more information from correlated traits and hence achieve higher prediction accuracy. The research first reported heritability estimation in rabbits by using genome-wide markers, and provided 50K as an optimal marker density for further microarray design, genetic evaluation and genomic selection in Angora rabbits. We expect that the work could provide strategies for GS in early selection, and optimize breeding programs in rabbits.

## 1 Introduction

The Angora rabbit is a well-known breed for fiber production that provides wool usually chosen for the production of luxury textile materials. Genetic improvement of wool production and quality is essential for achieving sustained increase in fiber production. Genomic selection (GS) is a potential breeding tool, and has been successfully employed in many farm animals, such as pigs and dairy cattle (Meuwissen et al., 2013; Gorjanc et al., 2015; Wiggans et al., 2017; Yang et al., 2020). GS can reduce the interval of generation, improve the accuracy and intensity of selection, and contribute to genetic improvement (He et al., 2019). A number of simulation and empirical studies on GS has realized impacts on improvement in the animal production (Solberg et al., 2008; Wiggans et al., 2017; Karimi et al., 2019; Yang et al., 2020), and GS has been effectively used in animal breeding programs for more than a decade (Hayes et al., 2009; Jannink et al., 2010). The exploitation of genome-assisted approaches could greatly benefit breeding efforts in Angora rabbits, though rabbits breeding is slower to adopt this technology. In rabbits, a high-density commercial SNP microarray (Affymetrix Axiom OrcunSNP Array, around 200k SNPs) was not available until 2015, and a lack in inexpensive chips and high genotyping cost by genome sequencing in rabbits delay genomic selection application; Additional issues such as the small economic value of paternal rabbits and the short generation interval are still limiting genomic selection as an evaluating method (Mancin et al., 2021).

Various factors appear to affect prediction accuracy in genomic selection (Covarrubias-Pazaran et al., 2018; Krishnappa et al., 2021). Marker density is a force driving the prediction accuracies of GS, and has been so far one of the most studied factors. It is suggested that high density markers can improve the prediction accuracy (Hickey et al., 2014; Al-Khudhair et al., 2021), and the consensus is that a higher number of markers usually yield higher accuracy reaching a plateau (Wang et al., 2017; Krishnappa et al., 2021). In the presence of genome resequencing, genome-wide SNPs are available for rabbits, but what density of markers is optimal for GS in Angora rabbits, *i.e.*, the density reaching a plateau, remains obscure, since the efficient SNP number could reduce the dimensionality of the GS model.

Various studies related to GS have been mostly confined to single trait in the recent past, although they performed not very well in cases of pleiotropy, missing data and low heritability (Boison et al., 2017; Budhlakoti et al., 2019). Gradually, studies were carried out to explore the possibility of methods for GS based on multiple traits that enabled to provide accurate genomic prediction by exploiting the information of correlated structure among response (Budhlakoti et al., 2019). In addition, breeders in animal breeding usually record one trait at multiple times throughout the lifetime of animals that are often strongly genetically correlated. The optimal estimation procedure is to combine information from multiple records of different traits or different times to obtain genomic estimated breeding values (GEBV) using the multi-trait models (Okeke et al., 2017; Covarrubias-Pazaran et al., 2018). In the breeding of Angora rabbits, we have very little idea about the performance of GS, so single-trait and multi-trait models should be explored.

In this study, we used the genomic resources of Angora rabbits in hand to test the usefulness of genomic selection. In order to maximize genomic prediction accuracy, we focused on estimating the optimal marker density undergoing a renaissance thanks to genome resequencing, and comparing the GS performance between single-trait and multi-trait models for genomic best linear unbiased prediction (GBLUP). The research would provide strategies for GS in early selection of wool production, and optimize breeding programs in Angora rabbits.

## 2 Materials and methods

### 2.1 Animal phenotypes and genotypes

A total of 629 Agora rabbits (298 males and 331 females) used for this study were from same batch. All rabbits were housed under the same conditions on a farm, including diet, water and temperature. In production practice, the rabbits are artificially inseminated with mixed semen, so there is not a definite pedigree information for the studied population. The wool is harvested around every 70 days from 70 days of age, and after the third shearing, the rabbits are selected for breeding. The associated wool traits including length of fine wool (LFW), diameter of fine wool (DFW), coefficient of variation of diameter of fine wool (CVDFW), length of coarse wool (LCW), rate of coarse wool (RCW) and weight of sheared wool (WSW) were measured at 70, 140 and 210 days of age. In addition, body weight (BW) was measured at the same days of age. The descriptive summary was provided for the traits in Supplementary Table S1.

Ear samples were collected from the individuals. Genomic DNA was isolated using the Qiagen MinElute Kit. Genomic DNA from each sample was used to construct a paired-end library (PE150) with an insert size of ∼350 bp. All libraries were sequenced on the DNBSEQ-T7 platform. By low-coverage whole genome sequencing (LCS), an average of 3.84X genomic coverage was sequenced, with the read depth varying from 1.51X to 8.03X. Read quality was assessed using FastQC (https://www.bioinformatics.babraham.ac.uk/projects/fastqc/). Adapters and low-quality bases were removed using Trimmomatic (Bolger et al., 2014). Sample reads were mapped to the rabbit reference sequence GCF_000003625.3 (*Oryctolagus cuniculus*) using BWA-mem (Li and Durbin, 2009). SNPs were called using BaseVar (Liu et al., 2018) and imputed genotype dosages at missing sites using STITCH (Davies et al., 2016). The SNPs were filtered for an imputation info score >0.4 using Bcftools (Li, 2011), and then with ‘MAF >0.05, genotype missing rate <0.1 and a Hardy-Weinberg equilibrium (HWE) *p*-value > 1E-6’ using PLINK (Chang et al., 2015). The sites which were missing in 10% of the individuals after STITCH imputation were then imputed by Beagle v5.1 Beagle v5.1 (Browning et al., 2018). A total of 18,577,154 high-quality SNPs (imputation accuracy above 98%) were used after stringent quality control. The manipulation of phenotypes and genotypes is detailed in the previous study (Wang et al., 2022).

### 2.2 Models

In our studies, univariate linear mixed models (uvLMM) were used to analyze the traits measured at three time points, respectively. The univariate linear mixed models are formulated as

y=Xb+Zu+e (1)

Here, y is the phenotypic vectors of a specific time point; b is the vector of fixed effects including population mean, batch and sex; u is the vector of random genetic effects; e is the vector of random residuals. X and Z are the corresponding design matrixes. The distributions of random effects are

$$u∼N\left(0,G\sigma_{u}^{2}\right),\;e∼N\left(0,\;I\sigma_{e}^{2}\right)$$ (2)

Where, $\sigma_{u}^{2}$ is the genetic variance; $\sigma_{e}^{2}$ is the residual variance; G is the genomic relationship matrix built with method of VanRaden (Vanraden, 2008); I an identity matrix.

To test the performance of GS using multivariate linear mixed models (mvLMM), we regarded the records from three time points of one trait as different traits and used mvLMM to analyze the data. The multivariate linear mixed models are formulated as

$$\left[\begin{array}{c} y_{1}\\ y_{2}\\ y_{3} \end{array}\right]=\left[\begin{array}{ccc} X_{1} & & \\ & X_{2} & \\ & & X_{3} \end{array}\right]\left[\begin{array}{c} b_{1}\\ b_{2}\\ b_{3} \end{array}\right]+\left[\begin{array}{ccc} Z_{1} & & \\ & Z_{2} & \\ & & Z_{3} \end{array}\right]\left[\begin{array}{c} u_{1}\\ u_{2}\\ u_{3} \end{array}\right]+\left[\begin{array}{c} e_{1}\\ e_{2}\\ e_{3} \end{array}\right]$$ (3)

All symbols have the same meaning with the single-trait models, and subscripts ( i=1,2,3 ) indicate the ith time point. The distributions of random effects are

$$\left[\begin{array}{c} u_{1}\\ u_{2}\\ u_{3} \end{array}\right]∼N\left(0,\;\sum_{u}⊗G\right),\;\left[\begin{array}{c} e_{1}\\ e_{2}\\ e_{3} \end{array}\right]∼N\left(0,\;\sum_{e}⊗I\right)$$ (4)

Where, $\sum_{u}$ and $\sum_{e}$ are a 3×3 covariance matrix for the genetic effects and residual errors.

### 2.3 Marker densities

To evaluate the influence of marker density on the heritability estimation and genomic prediction, we randomly selected 0.5K, 1K, 3K, 5K, 10K, 50K, 100K, 500K, 1M and 2M from the original 18.6M markers. Then, we used these randomly selected markers to build the genomic relationship matrix, and estimate heritabilities and genomic breeding values with univariate linear mixed models. For each marker density, we repeat this process for 30 times to obtain stable results.

### 2.4 Cross-validation

We used 10-fold cross-validation (CV) to assess the accuracy of the genomic prediction. The 629 individuals were randomly shuffled and split into 10 groups. One of them was used as a validation population in turn, and the remaining nine groups used as a training population. The accuracy of genomic prediction was assessed by the correlation between corrected phenotypic values ( $y_{c}$ ) and GEBVs in the validation population ( $r_{y_{c},GEBV}$ ). Here, the corrected phenotypic values were calculated with general linear model, which removed sex and batch effects from the original phenotypic values. For the three-trait models analysis, we also compared different leave-out strategies: 1) leave out the observations of all the three time points; 2) leave out the observations of the last two time points; 3) leave out the observations of the last time point. The aim of the three leave-out strategies was to explore whether and how the accuracy of the genomic prediction would be improved with early measured traits. In the study, we used two-sample *t*-test to determine whether the prediction accuracies from two experiments (varied marker densities or models) were significantly different from each other.

### 2.5 Implements

The genomic relationship matrix was built with GMAT (https://github.com/chaoning/GMAT), and uvLMM and mvLMM were implemented with DMU package (https://dmu.ghpc.au.dk/dmu/).

## 3 Results

### 3.1 50K markers are the baseline to estimate heritability for angora rabbits

**FIGURE 1:**
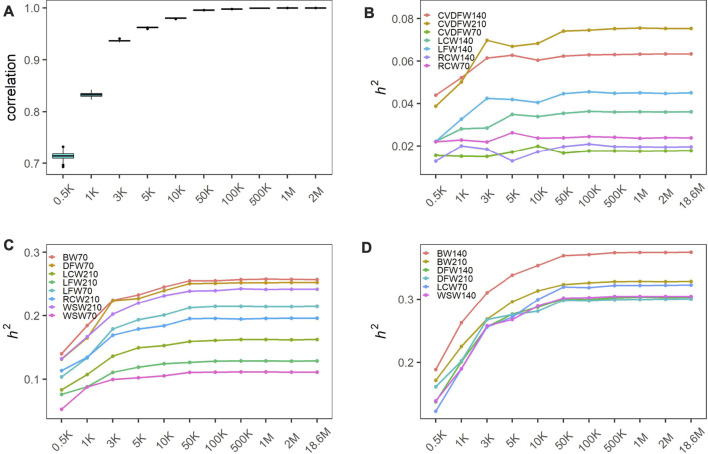
Heritability estimation of wool traits and body weight with varied marker densities. **(A)** Pearson correlation coefficients between all genomic relationship matrixes built from randomly selected markers; The changing estimated heritability for low heritability traits **(B)**, medium heritability traits **(C)** and high heritability traits **(D)**. Traits were categorized into three categories according to the heritability estimated with the original sequencing markers: low heritability (<0.1), medium heritability (0.1–0.3) and high heritability (>0.3).

In order to produce different marker densities, we randomly selected 0.5K, 1K, 3K, 5K, 10K, 50K, 100K, 500K, 1M and 2M from the original sequencing markers and repeat 30 times for each marker density to reduce the sampling error. We calculated the Pearson correlation coefficients between all genomic relationship matrixes built from randomly selected markers for each maker density. We found that the Pearson correlation coefficients increased rapidly and the dispersion degree decreased with the increase of marker density from 0.5K to 50K, and the values tended to be steady with the minimum value exceeding 0.99 (Figure 1A). We categorized traits into three categories according to the heritability estimated with the original sequencing markers: low heritability (<0.1), medium heritability (0.1–0.3) and high heritability (>0.3), and showed the average estimated heritability of 30 random selections for each marker density in Figures 1B–D. We observed that estimated heritabilities increased rapidly with the marker density increasing from 0.5K to 50K, and then maintained steady when the marker density continued to increase for all levels of heritability. The heritabilities estimated by the genome markers of 18.6M were listed in Table 1.

**TABLE 1:** Heritability estimated by the genome markers of 18.6M in the single-trait model.

Trait	Heritability	Trait	Heritability
BW70	0.257±0.05367	LFW70	0.214±0.0512
BW140	0.375±0.05271	LFW140	0.045±0.0385
BW210	0.328±0.05272	LFW210	0.129±0.04422
CVDFW70	0.018±0.03479	RCW70	0.024±0.03705
CVDFW140	0.063±0.03697	RCW140	0.02±0.03683
CVDFW210	0.075±0.04199	RCW210	0.196±0.04849
DFW70	0.252±0.04953	WSW70	0.111±0.04758
DFW140	0.303±0.05209	WSW140	0.305±0.05328
DFW210	0.301±0.05111	WSW210	0.242±0.0502
LCW70	0.323±0.05669		
LCW140	0.036±0.03552		
LCW210	0.162±0.04733		

### 3.2 50K markers can achieve ideal prediction accuracy for angora rabbits

**FIGURE 2:**
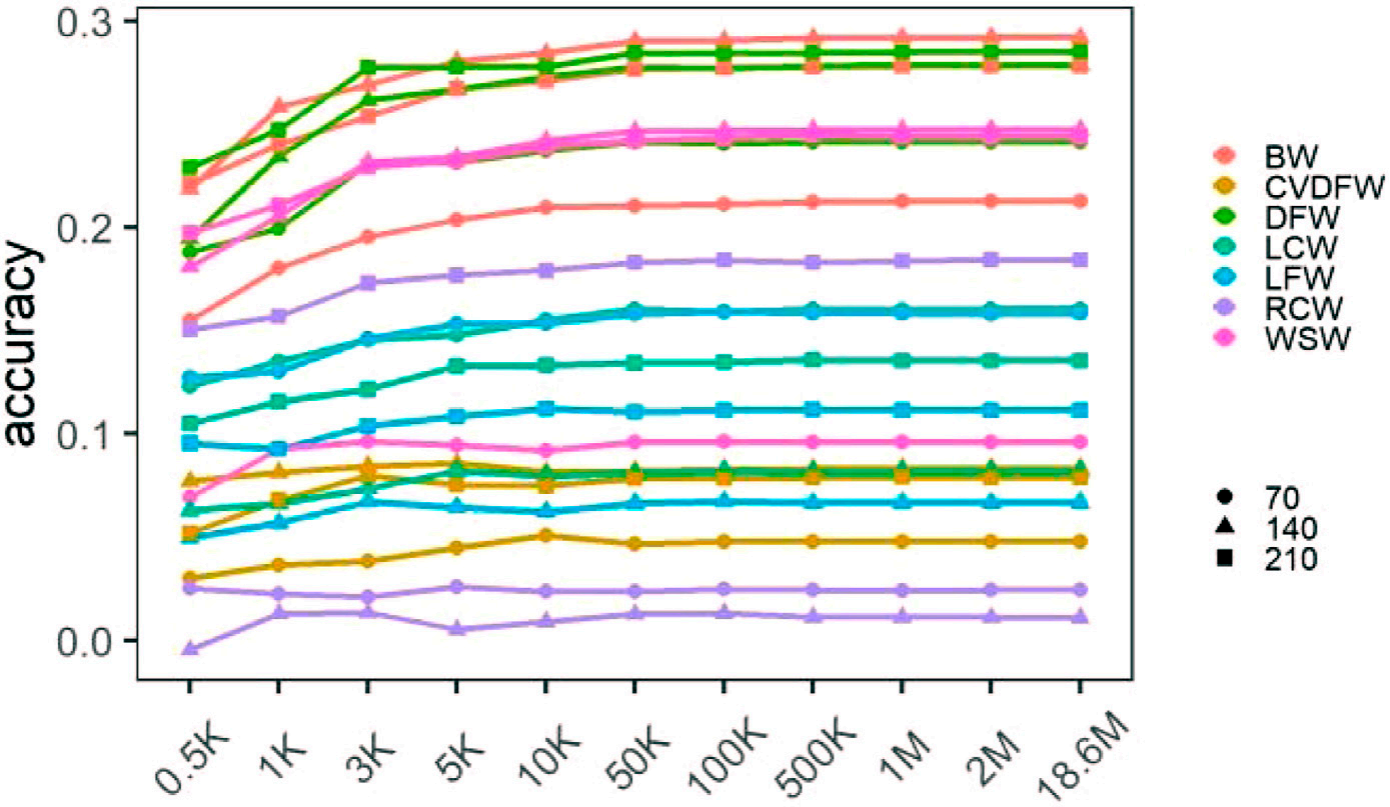
Mean prediction accuracies of cross-validation under different marker densities.

We calculated the prediction accuracy for each trait by averaging the cross-validation results of 30 random selections (Supplementary Table S2), and showed the changing prediction accuracy with the increase of marker density in Figure 2. For all traits, the mean accuracies were lower than 0.3 regardless of marker density. Similar to the change tendency of estimated heritability, we found that the prediction accuracy increased rapidly with the increase of marker density from 0.5K to 50K, and it improved very little when the marker density continued to increase. In addition, the significance of the differences between the prediction accuracies under different marker densities was listed in Supplementary Table S3. There was no significant difference between the accuracies under the marker density of 50K and 100K for all the traits, while when comparing 50K–10K, the difference between the accuracies was significant for several traits such as BW140, BW210, DFW210, LCW70 and WSW140.

### 3.3 Multiple-trait models can improve the prediction accuracy in genomic selection

**FIGURE 3:**
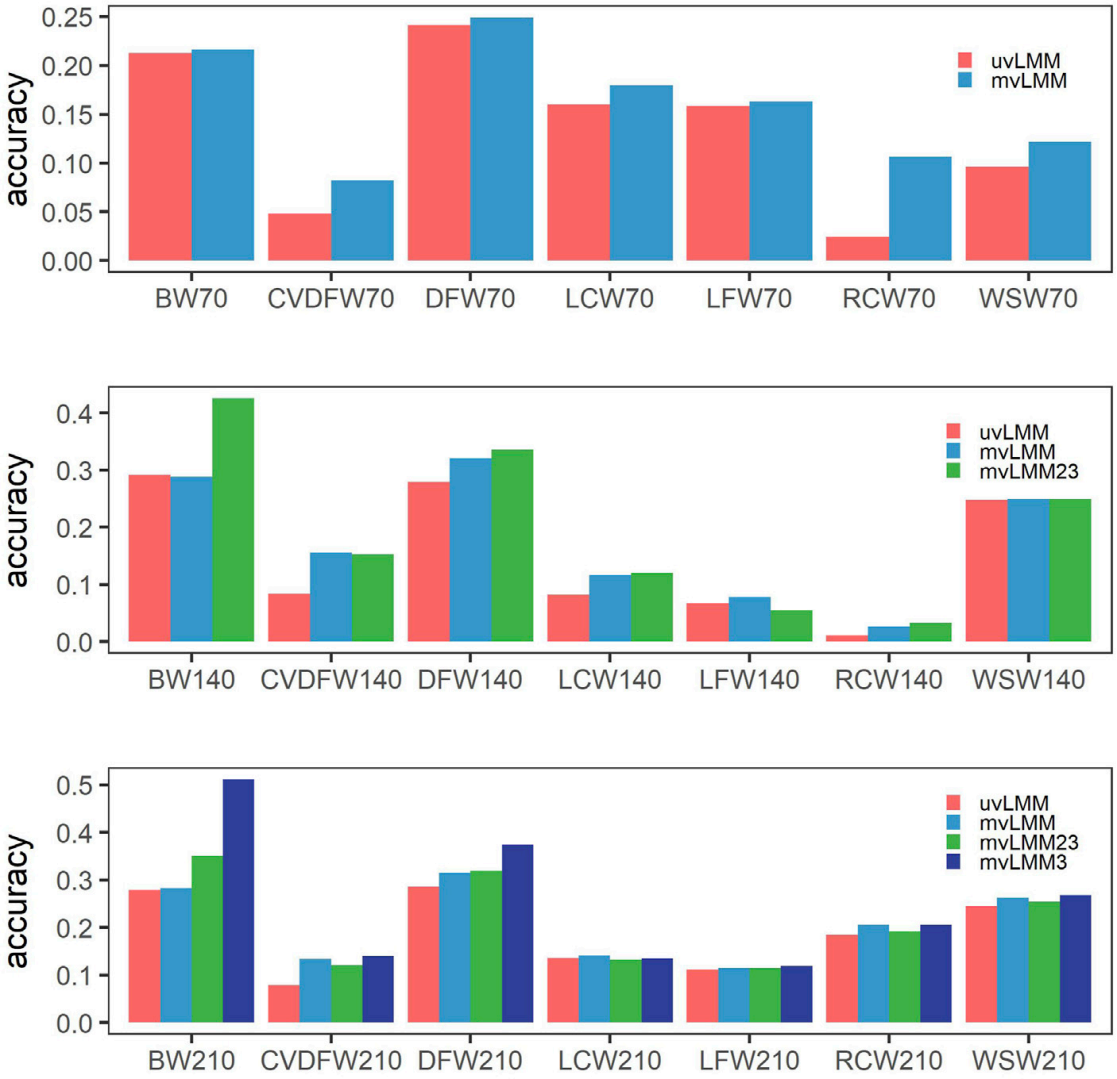
Mean prediction accuracies of cross-validation by different models. uvLMM: univariate linear mixed models which analyzed records of different time points, respectively; mvLMM: multivariate linear mixed models which considered the correlations of different time points and leave out the observations of all the three time points in the cross-validation experiments; mvLMM23: leave out the observations of the second and third time points in the cross-validation experiments; mvLMM3: leave out the observations of the third time points in the cross-validation experiments.

We applied the multiple-trait models to analyze the records from three time points of one trait. Compared to the single-trait models, the prediction accuracy of nearly all traits could be improved, except that it was slightly decreased for BW140 which was decreased from 0.292 from 0.288 (Figure 3, Supplementary Table S4 and S5). The Pearson correlation coefficient between the increased prediction accuracy from single-trait to multiple-trait models and estimated heritability was -0.584 (*p* = 0.0055), which indicated that the prediction accuracy of traits with lower heritability can be improved more with multiple-trait models. For example, CVDFW belonging to low heritability traits, its estimated heritabilities at three time points were 0.018, 0.063 and 0.075, respectively, but their prediction accuracy could be improved by 71.35%, 85.71% and 68.81%, respectively.

In the cross-validation experiments, if we kept the observations of early time points in the validation groups, the prediction accuracy could be further improved by multiple-trait models; and the more observations of early time points kept, the higher prediction accuracy could reach for the majority of traits (Figure 3 **,** Supplementary Table S4 and S5).

## 4 Discussion

Genomic selection promises to accelerate genetic gain in animal breeding programs (Meuwissen et al., 2013; Gorjanc et al., 2015; Yang et al., 2020). Practically, to implement GS in Angora rabbit breeding, it is necessary to evaluate different marker densities and GS models to develop suitable strategies for an optimized breeding pipeline.

Low-coverage sequencing combined with genotype imputation boosts the number of SNPs across the genome. It plays out advantages in obtaining genotyping information since both DNA library and sequencing cost decreased (Nicod et al., 2016; Meier et al., 2021) especially when lacking in microarray (Davies et al., 2016; Davies et al., 2021). In this study, we evaluated different marker densities for heritability estimation and genomic selection, and provided 50K as an optimal marker density for further microarray design, genetic evaluation and genomic selection in rabbits, since the efficient SNP number could reduce the dimensionality of the calculation model.

The heritability of various traits in rabbits was traditionally estimated by using pedigree information (Dige et al., 2012; El Nagar et al., 2020; Montes-Vergara et al., 2021). To our knowledge, this study was the first report for heritability estimation in rabbits by using genome-wide markers. What’s more, for Angora rabbits, little information is available on heritabilities of production performance and economic traits. The previous estimation using pedigree information included the heritability of wool production, coarse wool rate and body weight of Wan-strain Angora rabbits at 11-month-old (0.33, 0.21 and 0.43, respectively) (Zhao. et al., 2016), and the heritability of weaning weight, wool yield of first, second and third clips of Angora rabbits (0.24, 0.22, 0.20 and 0.21, respectively) (Niranjan et al., 2011). By exploring the influence of marker density on heritability estimation, we estimated stable heritabilities for wool and body weight traits of Angora rabbits at the marker density of 50K.

It becomes clear that the increase in the marker density by panels and even genome sequencing could not result in ceaselessly increase in the accuracy of genomic selection (Chang et al., 2019). In this study, the marker density showed major effects on the improvement of prediction accuracy below 50K, which showed the accuracy predicted by GS increased as the marker density increased for all traits in the rabbit population. However, above a threshold of 50K, the marker density showed minor effects. 50K is a density of genome markers in common usage for livestock genetics and breeding (Nandolo et al., 2019; He et al., 2020; Bhuiyan et al., 2021; Liu et al., 2021; Singh et al., 2021). Similar phenomenon was found in other species though the baseline of marker density was different (Spindel et al., 2015; Wang et al., 2017). The threshold where the plateau takes place might be associated with the extent of linkage disequilibrium (LD) between genome markers and QTLs. At a long extent of LD, the number of independent segments in the genome is expected to be small, which means fewer markers are needed to mark all segments (Wientjes et al., 2013; Wang et al., 2017). In the present study, the average pairwise LD r2 values decreased to 0.16 at 500 kb and to 0.11 at 1 Mb (Wang et al., 2022), and the population was considered to have a relatively slow decay of LD similar to other livestock population, hence 50K, a small number of markers, was sufficient to produce the accurate prediction.

A large number of genomic selection studies have focused on single-trait analyses (Boison et al., 2017; Budhlakoti et al., 2019). However, many traits are genetically correlated, such as the Angora wool traits among different shearing times. It has been shown that a multiple-trait genomic model had higher prediction accuracy than a single-trait genomic model, and the use of multiple-trait models is one of the ideas to increase the predictive ability of GS (Guo et al., 2014; Covarrubias-Pazaran et al., 2018). In this study, the majority of traits reached higher accuracy predicted by multiple-trait models than by single-trait models, because multiple-trait models used information from genetically correlated traits (Jia and Jannink, 2012). Furthermore, we observed high significant negative correlation between the increased prediction accuracy from single-trait to multiple-trait models and estimated heritability. The results indicated that low-heritability traits can borrow more information from correlated traits and hence achieve higher prediction accuracy. Especially, the prediction accuracy of BW140 with the highest heritability among the analyzed traits, was slightly decreased. Since many wool traits belong to medium and low heritability, this characteristic of multiple-trait could be very important in Angora rabbits breeding (Jia and Jannink, 2012).

## 5 Conclusion

Genomic selection was applied to Angora rabbits based on low-coverage sequencing combined with genotype imputation. A total of 18,577,154 high-quality SNPs were obtained with imputation accuracy above 98%. From the original markers, 0.5K, 1K, 3K, 5K, 10K, 50K, 100K, 500K, 1M and 2M were randomly selected and evaluated, resulting in 50K markers as the baseline for the heritability estimation and genomic prediction. Comparing to the GS performance of single-trait models, the prediction accuracy of nearly all traits could be improved by multi-trait models. Furthermore, we observed high significant negative correlation between the increased prediction accuracy from single-trait to multiple-trait models and estimated heritability. The results indicated that low-heritability traits could borrow more information from correlated traits and hence achieve higher prediction accuracy. The research first reported heritability estimation in rabbits by using genome-wide markers, and provided 50K as an optimal marker density for further microarray design, genetic evaluation and genomic selection in Angora rabbits. We expect that the work could provide strategies for early selection, and optimize breeding programs in rabbits.

## Data Availability

The datasets presented in this study can be found in online repositories. The names of the repository/repositories and accession number(s) can be found below: https://www.ncbi.nlm.nih.gov/, PRJNA810279.
